# 30th SESPO CONGRESS. Prevention leads dentistry. Madrid, 17–18 October 2025

**DOI:** 10.4317/jced.1122335667807

**Published:** 2026-02-01

Abstracts of the scientific communications follow.

