# Supplementary material for: 30th SESPO CONGRESS. Prevention leads dentistry. Madrid, 17–18 October 2025
Source: J Clin Exp Dent. 2026 Feb 1;18(Suppl 1):S1–S29. doi: 10.4317/jced.1122335667807 (PMC12861062; doi:10.4317/jced.1122335667807)
Supplement: Related texts

# Abstract: 01. UPDATE ON THE 'CEPILLÍN CEPILLÁN' PROGRAM: AN INTERSECTORAL APPROACH TO PROMOTING ORAL HEALTH IN EARLY CHILDHOOD EDUCATION IN THE CANARY ISLANDS

**Authors:** G. Gómez Santos, M.C. Siverio Delgado, J. Amaya González, J. Váquez García-Machiñena

**Affiliations:** 1 Dirección General de Salud Pública del Servicio Canario de la Salud, Santa Cruz de Tenerife; 2 CEO Guajara, Fasnia (Tenerife); 3 Dirección General de Programas Asistenciales del Servicio Canario de la Salud, Santa Cruz de Tenerife.

## Introduction

Cepillín Cepillán is an oral health intervention program aimed at the second cycle of Early Childhood Education (ages 3–5) in public schools across the Canary Islands. The program has been evolving since 2006.

## Objectives

To inform and train teachers, students, families, legal guardians, and school dining staff in healthy oral hygiene practices, helping to prevent oral diseases and promote a healthy lifestyle.

## Material and Methods

In 2025, collaborative work was conducted between the Directorate-General for Public Health of the Canary Islands Health Service and the Directorate-General for Curriculum Development, Inclusion, and Innovation of the Ministry of Education, Vocational Training, Physical Activity, and Sports. This collaboration, framed by the Joint Resolution of November 17, 2021, sought to update the program in accordance with current educational legislation. Educational materials were produced following these guidelines.

## Results

The resources created include: • A manual for the educational community. • Twelve workshops for classroom and home activities. • An integrative learning situation. • A teacher survey to assess the materials. • A self-paced online course to improve participants’ knowledge.

## Conclusions

Collaboration between the health and education sectors has strengthened intersectoral action, promoting health from early childhood. The program can contribute to preventing disease, improving academic performance, reducing social inequalities, supporting comprehensive child development, and fostering community participation.

# Abstract: 02. DESENSITISATION PROGRAMME FOR PATIENTS WITH LEVEL 3 AUTISM SPECTRUM DISORDER

**Authors:** M.M. Ruiz García, S. Junco García, K.E. Yarasca Arcos, J.L. Cabarcos Dopico

**Affiliations:** 1 C.S Daroca, Madrid; 2 CS Castroviejo, Madrid; 3 CS Pavones, Madrid; 4 Fundación Aucavi, Madrid.

## Introduction

Dental care for people with Autism Spectrum Disorder requires specific adaptations and desensitisation programmes, given their characteristics in coping with anxiety and complex environments.

## Case Report

This is a clinical case of a male patient who began the programme at the age of thirteen and attended for two years. He has level 3 Autism Spectrum Disorder, characterised by persistent deficiencies in communication and social interaction, anticipates routine situations, needs the help of pictograms and has severe difficulties in expressing his needs, resorting to self-harming behaviours when faced with frustration. The patient attended twice a week for two years for scheduled ten-minute appointments where gradual approaches to procedures and anticipations with individualised pictograms were used, collaborating with the educational and family environment. At the beginning of the sessions, he exhibited type I behaviour according to the Frankl scale, with uncooperative behaviours, but by the end of the sessions, he had achieved type IV behaviour.

## Conclusions

Through systematic desensitisation, the patient was able to receive comprehensive healthcare in order to undergo more complex treatments, avoiding restrictive measures and demonstrating its effectiveness not only in the dental field. It is necessary to implement desensitisation programmes for people with Autism Spectrum Disorder and dental specialisation, as the benefits obtained are objective and can be extrapolated to other healthcare settings.

# Abstract: 03. UPDATE ON ORAL HEALTH POLICY IN THE BASQUE COUNTRY

**Authors:** J. Rueda Garmendia

**Affiliations:** 1 Ambulatorio de Azpeitia, Azpeitia (Gipuzkoa).

## Introduction

The use of the Children´s Dental Health Programme (PADI in Spanish) has shown a clear downward trend over the last 10 years. Hence, Public Health experts has raised awareness of this problem to political representatives in the Basque Parliament, within the Health Commission.

## Objectives

To present the evolution of the use of the Child Dental Care Programme (CDCP) in the Basque Country over the last 10 years and establish possible ways to improve the use of services.

## Material and Methods

The evolution of the percentage of the total population served by the healthcare network from 2015 to 2024 has been analysed (data obtained from the CDCP annual reports). Analysis of oral health data obtained from the 5th Pediatric Dental Epidemiological Study of The Basque Country, at ages 7, 12, and 14.

## Results

- Average CDCP utilization rate has been 65% in the last 10 years. - Tooth decay prevalence in permanent dentition: 2.6%, 25.9%, and 33.4%. - DMFT index: 0.03, 0.50, and 0.78%. - Restoration index: 11.9%, 67.8%, and 78.6%.

## Conclusions

More than 35% of the population does not use the Children´s Dental Care Programme annually, and despite positive oral health data, the trend in CDCP use is declining. The Basque Parliament intends to include the promotion of oral health for The Basque Health Service´s Community Health strategy. It has also urged all Osakidetza Primary Care professionals to raise awareness about the importance of dentistry, and Osakidetza (The Basque Health Service) has been asked to proactively cite children for the CDCP.

# Abstract: 04. EVALUATION OF LIFESTYLE HABITS AMONG UNDERGRADUATE STUDENTS FROM DIFFERENT ACADEMIC PROGRAMS

**Authors:** M. Servera Fuster, I. Coll Campayo, P. López Safont N Riutord Sbert -

**Affiliations:** 1 Adema, Palma de Mallorca.

## Introduction

Lifestyle plays a crucial role in public health. The university period is critical for establishing healthy habits. Assessing these behaviors across academic programs can help identify specific needs for health promotion.

## Objectives

To identify healthy habits, health-related behaviors, and risk factors among students in Dentistry, Nutrition, and Fine Arts, and to explore differences between programs.

## Material and Methods

Cross-sectional descriptive study using a self-administered questionnaire adapted from validated tools (WHO, ENALIA). Representative stratified sample (n=254). Variables include physical activity, oral health, dietary intake, screen time, tobacco and alcohol use. Statistical analysis will involve chi-square tests and measures of association. The project has been approved by the Ethics Committee of the Illes Balears (registration IB 5691/24 PI).

## Results

The questionnaire has been validated and administered, with high student engagement. So far, 164 questionnaires have been collected, with a total of 254 expected. Data collection and statistical analysis are currently underway.

## Conclusions

This study will enable the identification of differences across academic programs and support the development of targeted health promotion strategies in the university setting. Final results will be presented at the congress.

# Abstract: 05. STUDY ON SATISFACTION AND QUALITY OF LIFE IN VULNERABLE EDENTULOUS OR PARTIALLY EDENTULOUS INDIVIDUALS: RESEARCH PROTOCOL

**Authors:** I.1. Coll Campayo, A.2. Jaume Campomar, M.A.3. Casado Mac Donald, C.4. Morey Vázquez, C.4. Lluy González

**Affiliations:** 1 Adema, Palma; 2 Jaume Dentistes, Pollença; 3 Dentipalma, Palma; 4 Clínica Solidaria DSR, Palma

## Introduction

Edentulism, whether partial or complete, negatively affects oral, functional, and social quality of life, especially among vulnerable populations. Proper prosthetic rehabilitation can significantly improve these outcomes.

## Objectives

To compare the effectiveness of conventional dentures versus implant-retained prostheses in improving oral and social quality of life in vulnerable edentulous or partially edentulous individuals.

## Material and Methods

This is an observational, retrospective, case-control study. The sample will consist of 100 patients from the Solidarity Clinic. The validated OHIP-20sp questionnaire will be administered before treatment, and the OHIP-14post at one, six, and twelve months after rehabilitation. Clinical, functional, aesthetic, and social variables will be analyzed. The project has been approved by the Research Ethics Committee (CEIB: IB5561/24PI). The study has not yet started; this presentation will focus on the methodological protocol.

## Results

No results are available yet, as the study has not begun.

## Conclusions

This presentation will detail the methodological planning of a study designed to provide evidence on the impact of different prosthetic treatments on oral quality of life in vulnerable populations.

# Abstract: 06. SURVEY ON KNOWLEDGE, IMPLEMENTATION, AND PERCEPTION OF PREVENTIVE AND MINIMAL INTERVENTION PROTOCOLS FOR DENTAL CARIES AMONG DENTISTS IN PRIVATE CLINICS IN MADRID

**Authors:** M. De Feo Tolosa, D. González Alarcón, M.V. Mateos Moreno, M.R. Garcillán Izquierdo

**Affiliations:** 1 UCM, Madrid; 2 UFV, Madrid

## Introduction

Dental caries is a major oral health problem. Evidence-based protocols such as CAMBRA, ICDAS, and CariesCare 4D exist, but their adoption in private dental clinics in Madrid remains unclear.

## Objectives

To assess knowledge and implementation of preventive caries protocols, identify barriers, and analyze dentists’ perceptions of preventive dentistry.

## Material and Methods

An observational, cross-sectional, descriptive study was conducted. A structured survey was administered to dentists from nine private clinics in Madrid, selected through stratified sampling. Sociodemographic, knowledge, clinical application, and perception variables were collected. Descriptive statistics and frequency tables were used for analysis.

## Results

Eighty-nine percent of dentists reported familiarity with ICDAS, 100% with non-operative techniques, and 56% with CAMBRA. Nevertheless, only 33% reported using CAMBRA regularly and 44% ICDAS. The most cited barriers were lack of patient interest, low profitability, and insufficient training. All respondents considered additional training necessary, and most expressed interest in receiving it.

## Conclusions

A clear gap exists between knowledge of preventive protocols and their clinical implementation. Although dentists acknowledge the importance of prevention, economic and organizational barriers limit its use. The strong demand for training highlights an opportunity to strengthen the role of preventive dentistry in private practice.

# Abstract: 07. LEVEL OF KNOWLEDGE ABOUT SUSTAINABILITY AND THE SDGS AMONG UNDERGRADUATE DENTISTRY STUDENTS

**Authors:** Y.H. Qiu Zhu, J.M. Almerich Silla, M. Puig Silla, O. Zurriaga Llorens, T. Almerich Torres

**Affiliations:** 1 Facultat de Medicina i Odontologia, Universitat de València, Valencia.

## Introduction

The Sustainable Development Goals (SDGs) outlined in the 2030 Agenda aim to ensure the well-being and proper development of present and future generations. The key role played by universities in the education of future professionals highlights the importance of integrating sustainability into higher education.

## Objectives

To assess the level of knowledge and awareness of sustainability and the SDGs among undergraduate Dentistry students at the University of Valencia.

## Material and Methods

A validated questionnaire was distributed to third, fourth, and fifth-grade students via the LimeSurvey platform. Descriptive and inferential statistical analyses were conducted, including Student's t-test, ANOVA, and Cronbach’s alpha (α = 0.05).

## Results

A high response rate was obtained, n = 165 (84.74%), with strong statistical reliability (Cronbach’s alpha 0.92 and 0.95). The overall level of knowledge was low across the sample. Statistically significant differences were observed between genders in numerous items, with male students scoring higher. Differences were found, not as significantly, among academic years, with third-year students reporting higher perceived knowledge of the SDGs.

## Conclusions

Undergraduate Dentistry students at the University of Valencia demonstrate a low level of knowledge regarding sustainability and the SDGs, although they show greater familiarity with health-related goals. These findings highlight the need to incorporate more sustainability-related content into the curriculum to enhance the academic training of future professionals.

# Abstract: 08. RELATIONSHIP BETWEEN ORAL MICROBIOTA AND ALZHEIMER’S DISEASE

**Authors:** D. García Villalón, S. Hernández López, P. Hernández López, V. García Villalón

**Affiliations:** 1 Clínica dental Dra. Silvia Hernández López, Linares.

## Introduction

Alzheimer’s disease is a progressive neurodegenerative disorder that affects cognitive functions such as reasoning, learning, thinking, memory, and language. This research claims there is a link between Alzheimer’s disease and the oral microbiota, as a state of dysbiosis will lead to oral diseases and promote the growth of bacteria harmful to the nervous system, which are able to create a toxic and inflammatory environment.

## Objectives

The main goals are to establish Alzheimer’s symptoms, to explain the functions of oral microbiota, to analyse its link to Alzheimer’s disease, to identify harmful bacteria to the nervous system and to highlight the importance of having good oral hygiene in order to prevent neurodegenerative diseases.

## Material and Methods

A literature review was conducted in PubMed and in Google Scholar in May 2025, using terms related to oral microbiota and Alzheimer’s disease. Articles in English and Spanish in the last ten years were included, prioritizing original studies, systematic reviews and meta-analysis. This selection was done manually, excluding duplicate or those with limitations in scientific rigour.

## Results

A balanced oral microbiota protects the oral cavity from pathogenic agents. In a state of dysbiosis, diseases such as Gingivitis and periodontitis may arise, leading to the proliferation of Porphyromonas gingivalis and Treponema denticola, bacteria associated with Alzheimer’s disease due to their inflammatory effects and neuronal damage they cause.

## Conclusions

There is a strong connection between oral microbiota and Alzheimer’s disease. Maintaining balanced oral microbiota through good oral hygiene can help prevent periodontal and neurodegenerative diseases.

# Abstract: 09. ANTIBIOTIC USE AMONG DENTAL STUDENTS: A CROSS-SECTIONAL STUDY

**Authors:** M. Mulero De Caso, S. Veiga Nieves, M. Zapata Cachafeiro, P. Fernández Rivero, C. García Seijas, A. Rodríguez Fernández

**Affiliations:** 1 Universidad de Santiago de Compostela, Santiago de Compostela.

## Objectives

(i) to determine the knowledge and attitudes of dental students on the use of antibiotics; (ii) to describe the training received and the sources of information on antibiotics used by students.

## Material and Methods

A cross-sectional study was conducted among undergraduate dental students studying at a Spanish university. Data were collected using a self-completed online questionnaire, which was distributed through social networks and messaging groups. Information was collected on knowledge, attitudes, academic background, sources of information and socio-demographic data.

## Results

249 students participated, 76.1% of whom were female. The active ingredients of choice were amoxicillin alone (55%) or with clavulanic acid (42.7%) in non-allergic patients and clindamycin (63.3%) or azithromycin (21.6%) in allergic patients. 12.2% would prescribe antibiotics in case of irreversible pulpitis, 47.3% in necrotising gingivitis and 31.5% to prevent infections after exodontia. Pharmacology and supervised clinical practice are the subjects in which they report having received the most training.

## Conclusions

The results of our study suggest that the knowledge of dental students is not adequate. Therefore, it would be advisable to reorient the theoretical contents of the degree to reinforce their knowledge during the undergraduate stage.

# Abstract: 10. SERVICE-LEARNING IN HEALTHY EATING. STUDENTS OF HEALTH SCIENCES AT THE UNIVERSITY OF VALENCIA

**Authors:** M. Puig Silla, A. Ruiz Miravet, J.M. Almerich Silla, J.M. Soriano Del Castillo, J.E. Iranzo Cortés, T. Almerich Torres

**Affiliations:** 1 Universitat de València, Valencia.

## Introduction

Service-learning (SL) is an educational methodology that combines academic learning with active participation in projects aimed at improving the community. The consumption of added sugars is a key risk factor in the development of non-communicable diseases (NCDs).

## Objectives

To assess the level of knowledge regarding healthy eating among Health Sciences students at the University of Valencia (UV). To encourage the involvement of Dentistry students in Service-Learning alongside students from other Health Sciences degrees.

## Material and Methods

A survey was designed to evaluate the level of knowledge on healthy eating among Health Sciences students at the University of Valencia. The survey was distributed by students of Preventive and Community Dentistry I to peers enrolled in other Health Sciences programs. Afterwards, they shared educational material on Healthy Eating.

## Results

A total of 161 surveys were collected from 11 degree programs. The average score was 6.93 (SD 1.89) out of 10. Students in Nutrition and Food Technology showed a higher level of knowledge compared to those in degree programs such as Speech Therapy, Nursing, or Psychology, which exhibited a lower level of knowledge.

## Conclusions

The knowledge demonstrated by the surveyed students can be improved, especially considering they are enrolled in health-related programs. Conducting the fieldwork has motivated Dentistry students to strengthen their understanding of healthy eating.

# Abstract: 11. SYSTEMATIC REVIEW OF DENTAL PRESCRIPTIONS: DATA SOURCES AND MOST FREQUENT EDICATIONS

**Authors:** C. García Seijas, E. Leite De Freitas, A. Rodríguez Fernández, M. Zapata Cachafeiro, M. Mulero De Caso, A. Figueiras Guzmán

**Affiliations:** 1 Universidade de Santiago de Compostela, Santiago de Compostela.

## Introduction

Studies suggest that analgesics and antibiotics are the most commonly prescribed drugs in dentistry. This paper reviews the methodology used to collect data on prescriptions in dentistry and the reliability of this information in various countries.

## Objectives

To describe the data collection methodology used in studies evaluating the prescription of drugs by dentists and to analyze which drugs are prescribed in dentistry.

## Material and Methods

A systematic review of articles published in the last 10 years in Spanish, English, and Portuguese was conducted. The search was conducted in the MEDLINE, Web of Science, and EMBASE databases. The following keywords were used: dentist, prescription, drugs, and dental pathology. Finally, a summary of the findings was carried out following the PRISMA methodology (CRD420251008402).

## Results

A total of 10,090 records were identified. After reviewing the titles and abstracts, 188 articles were read in full, of which 91 were included. Of these, 48 refer to analgesics, 46 to antibiotics, and 3 to anxiolytics. The records come from 23 different countries, mainly the United States.

## Conclusions

This review indicates that national registries are the main source of data on dental prescribing, providing reliability to studies. Antibiotics and analgesics are the most commonly prescribed drugs, reflecting the main clinical needs in dental practice. These results highlight the importance of promoting evidence-based clinical decisions and their impact on public health.

# Abstract: 12. PREVENTION OF MALOCLUSSIONS DUE TO PACIFIER USE

**Authors:** E. Sánchez Moreno

**Affiliations:** 1 Estudiante de quinto curso en Universidad Complutense de Madrid, Madrid.

## Introduction

Pacifiers are widely used in children. They can have benefits such as a calming effect and even analgesic properties in newborns, and are useful in preventig sudden infant death syndrome, but prolonged use can cause harmful oral effects.

## Objectives

To synthesize the scientific evidence regarding its effects on the development of malocclusions and their prevention through the interception of this non-nutritive habit.

## Material and Methods

Consultation of PubMed, Scopus, Cochrane Library and Dialnet databases, using the terms pacifier AND (anterior openbite OR crossbite), from July 2015 to July 2025, in English or Spanish, open access, and in humans aged 0 to 12 years.

## Results

 41 articles were obtained. According to scientific evidence, prolonged pacifier use can cause alterations in the swallowing pattern, a higher prevalence of anterior open bite and increased overjet (which may act as predisposing factors for dental trauma) and a decrease in intercanine distance. It can also interfere with breastfeeding.

## Conclusions

The duration and frequency of sucking can be decisive factors, increasing the likelihood of developing malocclusions. Pacifier use is associated with posterior crossbite, especially if it continues for a year or more. Preventing such malocclusions may also prevent possible dental trauma. It is recommended to limit their use, encourage breastfeeding and use physiologically designed pacifiers (rather than conventional ones). More well-designed, high-quality randomized clinical trials are needed regarding these recommendations.

# Abstract: 13. HARMLESS? E-CIGARETTES AND THEIR TRUE EFFECTS ON ORAL HEALTH

**Authors:** K. Rodriguez Masullo

**Affiliations:** 1 Complutense, Madrid.

## Introduction

While conventional tobacco is clearly associated with serious health risks, evidence regarding the effects of electronic cigarettes on oral health remains limited. These devices produce aerosols containing nicotine, flavorings, and potentially toxic compounds that may adversely affect the oral cavity.

## Objectives

To review the current literature on the effects of electronic cigarettes on oral health, with the aim of synthesising the available evidence and highlighting its clinical implications.

## Material and Methods

A systematic search was conducted in PubMed, Web of Science, and Scopus, limited to the past five years, using open-access filters and selecting studies classified as systematic reviews or meta-analyses. After removing duplicates, 17 articles were selected for detailed analysis.

## Results

The studies indicate that the use of electronic cigarettes is associated with an increased risk of periodontitis compared to non-smokers, although this risk is lower than that observed in conventional smokers. Additionally, alterations in the oral mucosa and oral microbiome have been identified, characterized by an increase in opportunistic bacteria and a reduction in salivary antimicrobial proteins. Furthermore, changes in salivary composition, elevated markers of cellular damage, and increased bacterial adhesion to the enamel surface have been documented-factors that contribute to the development of dental caries.

## Conclusions

Although vaping is perceived as less harmful than smoking tobacco, the evidence suggests that it poses significant adverse effects on oral health. There is a need to promote longitudinal studies with standardized methodologies to clarify its long-term impacts.

# Abstract: 14. RECRUITMENT OF CHILDREN IN PRIMARY CARE CONSULTATIONS IN CASTILLA-LA MANCHA

**Authors:** F. Gil Moreno

**Affiliations:** 1 CEDT Azuqueca de Henares, Azuqueca de Henares.

## Introduction

The Ministry of Health and Consumer Affairs, in its 2025–2027 Primary and Community Care Action Plan, has set the objective that 70% of children should visit their Oral Health Unit (USBD) at least once per year.

## Objectives

To analyze the results of child recruitment in the region of Castilla-La Mancha across the various primary care management areas, and to relate them to the number of Oral Health Units available. Are more children reached where there are more Oral Health Units?

## Material and Methods

Since 2022, SESCAM has published data on the percentage of children under the age of 16 who attend primary care consultations. The data published by each of the 14 management areas in Castilla-La Mancha for the years 2022–2024 were analyzed.

## Results

The 2022 results show that some management areas with fewer Oral Health Units actually reach a higher number of children. The average number of children seen per management area is also presented, revealing significant differences between the area with the highest number of children per unit (1,011 children/unit) and the one with the lowest (416 children/unit).

## Conclusions

Currently, only 20% of children in Castilla-La Mancha undergo an annual oral health check-up. There is no directly proportional relationship between human resources and recruitment rates. The data indicate a significant potential for improvement in most of the Oral Health Units (USBD) in our region.

# Abstract: 15. PIT AND FISSURE SEALANTS: WHAT FACTORS INFLUENCE SUCCESS?

**Authors:** S. Grano De Oro Saavedra, B. Cuesta Paniagua, I. Carrasco Jurado, G. Saavedra Marbán

**Affiliations:** 1 Universidad Complutense de Madrid, Madrid.

## Introduction

Sealants are a widely used treatment in caries prevention. The application of these materials allows the mechanical sealing of pits and fissures, preventing the accumulation of food debris and microorganisms. The correct retention of this material on the tooth«s surface and the abscence of filtrations is essential for them to be effective.

## Objectives

To analyse different factors that may influence good sealant retention on the tooth surface.

## Material and Methods

Literature searches were performed in PubMed, Google Scholar, and Cochrane using the keywords ‘sealant’ AND ‘pits and fissures.’ The inclusion criteria were: articles published in the last five years, whose full text was available free of charge in English and/or Spanish.

## Results

There are no studies that ensure that factors such as the type of isolation used, relative or absolute, or the composition of the sealants significantly influence better sealant integrity. Several authors have pointed out that the use of sodium hypochlorite as a deproteinising agent prior to the application of adhesive procedures, as well as the incorporation of adhesive systems, could improve the retention of sealants, as well as their clinical efficacy.

## Conclusions

Current evidence suggests that the use of deproteinising agents and adhesive systems improves stability over time, thereby influencing the success of sealants. However, it has not been shown that either the type of isolation or the composition of the sealant material affects the durability of thesealants. Further studies are needed to identify emerging improvements so that they can be incorporated into clinical practice.

# Abstract: 16. KNOWLEDGE, ATTITUDES AND PRACTICES REGARDING THE USE OF ANTIBIOTICS IN DENTISTRY. A QUALITATIVE STUDY IN CHILE

**Authors:** A. Juárez Membreño, A. Rodríguez Fernández, M. Zapata Cachafeiro, O. Vázquez Cancela, A. Prieto Campo, A. Figueiras Guzmán

**Affiliations:** 1 Universidad de Santiago de Compostela, Santiago de Compostela; 2 Hospital Clínico Universitario de Santiago de Compostela, Santiago de Compostela;; 3 Fundación Pœblica Galega de Investigación Biomédica Galicia Sur, Vigo.

## Introduction

Antibiotic resistance is a public health problem and is considered one of the greatest threats to global health. The inappropriate use of antibiotics is considered one of the main causes of microbial resistance.

## Objectives

To analyse knowledge, attitudes and practices regarding the prescription of antibiotics in dentistry.

## Material and Methods

A qualitative study was conducted using the focus group (FG) method targeting dentists working in Chile. The sample was selected through key informants and convenience sampling. To be part of the groups, it was necessary to: (i) be a graduate in dentistry; and (ii) be practising as a dentist at the time of the study. The meetings were organised through Microsoft Teams. Thematic analysis was used to identify relevant aspects of the transcripts.

## Results

Five FGs were conducted, involving 27 dentists. It was identified that complacency, external responsibility, healthcare burden, patient pressure, and cultural and social problems could mediate antibiotic prescribing. Participants pointed out that having adequate knowledge about the use of antibiotics improves the quality of prescriptions and provides arguments to combat patient pressure.

## Conclusions

Our results suggest that the lack of compliance with the regulation of antibiotic sales is a factor that may be increasing the culture of trivialising their use.

# Abstract: 17. ORAL AND SEXUAL HEALTH: IS PREVENTION THE BEST PROTECTION?

**Authors:** S. Ruiz Tabero, C. Hernández Garcés

**Affiliations:** 1 Clínica Complutense, Madrid.

## Introduction

Sexually transmitted diseases are infections with multiple etiologies that, due to their high incidence, constitute a major global public health problem. In addition to their systemic impact, they can appear in the oral cavity, making the dentist a key professional in early detection. Among the most relevant are the Human Papilloma Virus (warty lesions and oropharyngeal cancer), syphilis (oral ulcers) and the Human Immunodeficiency Virus (ulcers and secondary infections).

## Objectives

To inform and raise awareness about the impact of sexually transmitted diseases on oral health, as well as to evaluate the effectiveness of diagnostic criteria and available prevention methods.

## Material and Methods

A bibliographical review was carried out in PubMed and Scielo, limiting the search to the last ten years, articles of free access and studies of type systematic review and meta-analysis. Seventeen articles were obtained, of which 10 were selected for analysis.

## Results

The effectiveness of diagnostic methods in sexual diseases with oral and systemic impact, such as syphilis and human immunodeficiency virus, varies according to technique, clinical stage and social context. However, there is evidence that early diagnosis requires sexual anamnesis and oral examination, as well as medical interconsultation and follow-up, complemented by education and awareness of patients and professionals.

## Conclusions

Early diagnosis and education allow an effective approach to sexually transmitted diseases with oral manifestations. The active involvement of the dentist is essential for comprehensive detection, prevention and control.

# Abstract: 18. ORAL HEALTH IN THE INMATE POPULATION OF SANGMELIMA PRISON, SOUTHERN CAMEROON

**Authors:** A.N. Mankongo, J. Dongmo Bachelard, M. Lamas Oliveira, J. Sánchez Ituarte, A. Castaño Seiquer, D. González Alarcón

**Affiliations:** 1 Universidad de Sevilla, Sevilla; 2 Circular Research, Yaundé Camerœn; 3 Universidad Francisco de Vitoria, Madrid; 4 Universidad Europea de Madrid. Universidad de Nebrija, Madrid; 5 Circular Research. Universidad Francisco de Vitoria. Universidad de Nebrija, Madrid. Yaundé.

## Introduction

The public health system in Cameroon does not provide dental care for the prison population, despite the significant impact of oral diseases on the overall disease burden and quality of life.

## Objectives

To assess the oral health status of inmates at Sangmelima prison, identifying clinical and educational needs.

## Material and Methods

A mixed-methods study: quantitative (cross-sectional descriptive) and qualitative. The sample included 222 inmates (98.6% men; median age: 29.5 years), over a 12-month period. Open interviews and surveys were conducted following WHO methodology for dental caries and CPITN for periodontal deseases, with high inter-examiner agreement (kappa = 0.85).

## Results

The prevalence of dental caries was 69.4%, with a mean DMFT index of 2.99, which increased significantly with age (p = 0.002). Decayed teeth predominated (74.8%), followed by missing (24.9%) and filled teeth (0.3%). The restoration index was 0.3%. Of the 1,332 periodontal sextants examined, the most frequent were healthy (32.1%) and those with calculus (30.4%), with deep pockets mainly observed in older inmates. A total of 45 % required oral hygiene education and prophylaxis.

## Conclusions

The high burden of oral disease among the prison population demands priority attention. It is urgent to develop a specific agenda that promotes a comprehensive oral health action plan within the Cameroonian penitentiary system.

# Abstract: 19. SOLITARY MEDIAN MAXILLARY INCISOR SYNDROME

**Authors:** A. Ministral Masgrau, M. Pulido Martí, J. Masdevall Galter, E. Algans Escapa, M. Parés Roda, H. Cebriá Fondevila

**Affiliations:** 1 CAP Sarriá de Ter, Sarriá de Ter (Girona); 2 CAP Ripoll, Ripoll (Girona); 3 CAP Perelada, Perelada (Girona).

## Introduction

Single maxillary central incisor syndrome (SMI) is characterized by the presence of only one deciduous maxillary central incisor and one permanent . It can occur in isolation or in conjunction with other disorders of varying complexity (growth hormone deficiency, choanal atresia, holoproencephaly,...). It is an autosomal dominant inherited condition. It develops at 35-38 days of intrauterine life, and its incidence is 1 in 50,000 live births.

## Case Report

A 21-month-old boy presented with a single deciduous maxillary central incisor, centered in the midline and with no labial frenulum. The pediatrician referred him at 9 months due to an alveolar ridge injury following trauma. Peculiar anatomy was already observed even though no maxillary teeth were yet present. The periapical radiograph showed a single central tooth. We reviewed the scientific literature to better address the case and inform the family. Multidisciplinary workup and management are required to rule out other midline abnormalities or general involvement. Genetic testing is suggested.

## Conclusions

SICMU may be an isolated anomaly or accompanied by other more serious midline abnormalities and/or general involvement. The first visit in early childhood is important, not only to assess caries risk, but also to assess eruptive changes and anomalies that may occur. Coordination with the pediatric team is essential. We must provide parents with timely information, support, and follow-up, especially for therapeutic decisions regarding permanent dentition.

# Abstract: 20. ARTIFICIAL INTELLIGENCE IN PREVENTIVE DENTISTRY: INNOVATIONS IN EARLY DIAGNOSIS AND PERSONALIZED CARE

**Authors:** N. Toledano, J. Sánchez Ituarte

**Affiliations:** 1 Universidad Europea de Madrid, Villaviciosa de Odón, Madrid.

## Introduction

Currently, only 25–30% of dentists in Spain integrate artificial intelligence (AI) into their daily practice, despite over 50% of the population being familiar with this technology. Artificial intelligence (AI) is revolutionizing preventive dentistry by enhancing early diagnosis and personalized care.

## Objectives

This study evaluates AI’s impact on diagnostics, treatment planning, and patient care.

## Material and Methods

A systematic review was conducted, searching PubMed, Scopus, and Web of Science for articles from 2020 to 2025. The strategy used keywords (Òartificial intelligence,Ó Òdentistry,Ó Òprevention,Ó ÒdiagnosisÓ) and Boolean operators (AND/OR), filtering for peer-reviewed studies in English or Spanish on AI applications in caries, gingivitis, and periodontitis prevention. Fifteen articles were included after screening for relevance and quality, with risk of bias assessed using the ROBINS-I tool, noting low to moderate bias in most studies, though some lacked clear reporting of data sources.

## Results

The studyshow AI improves diagnostic accuracy by 20–30%, from 60–80% to 85–99%, with specificity above 90%, reducing unnecessary treatments and biological harm. Chatbots like ChatGPT-4 provide reliable oral hygiene guidance, while AI-based selfie apps significantly reduce plaque and gingivitis indices short-term. Ethical challenges, including data privacy and algorithmic bias, persist, alongside limited dentist training.

## Conclusions

AI fosters proactive prevention through precise diagnostics, tailored education, and personalized treatment plans, with potential for early oral cancer detection. Ethical integration and enhanced training are essential to maximize AI’s impact on oral health, particularly as only 25–30% of Spanish dentists adopt AI, despite over 50% of the population’s familiarity.

# Abstract: 01. PROTOCOL FOR REMINERALIZATION OF INCIPIENT CARIES LESIONS USING PREVENTIVE TREATMENTS IN PRIMARY CARE DENTISTRY

**Authors:** G. García Vicent, A.L. Lenguas Silva, M.V. Mateos Moreno, J.F. Martín Morales, M. De Feo Tolosa, M.R. Garcillan Izquierdo

**Affiliations:** 1 Facultad de Odontología (U.C.M.), Madrid.

## Introduction

Incipient caries lesions are frequently observed in Primary Care Dentistry.

## Objectives

To assess the effectiveness of a preventive remineralization treatment based on fluoride varnish.

## Material and Methods

A clinical trial was conducted at the Centro de Salud Mejorada del Campo of the Madrid Health Service, approved by the Ethics Committee of the Hospital Cl’nico San Carlos, in 35 patients between the ages of 8 and 19 with at least one early non-cavitated lesion (ICDAS I and II) and who had not received professional fluoride application in the previous 6 months. The lesions were re-evaluated every 3 months using laser fluorescence (DIAGNOdent¨ Pen 2190), after cleaning and drying the tooth surface. Three groups: 1. fluoride varnish with 22,600 ppm fluoride ion every 3 months combined with toothpaste with 1,450 ppm fluoride ion twice daily; 2. weekly application of fluoride varnish for 3 consecutive weeks every 3 months together with toothpaste; and 3. control group with toothpaste only.

## Results

The application of fluoride varnish for 3 consecutive weeks every 3 months yielded better results than the group every 3 months (p=0.003) and the control group (p=0.0495). ANOVA test (p<0.05 statistically significant).

## Conclusions

A white spot lesion remineralization protocol based on the use of fluoride varnish with 22,600 ppm fluoride ion for 3 consecutive weeks every 3 months should be implemented in oral health programs, providing a noninvasive, medical approach to caries disease.

# Abstract: 02. RELATIONSHIP BETWEEN THE TERRITORIAL SOCIOECONOMIC INDEX AND THE PERCENTAGE OF 7-YEAR-OLD CHILDREN AT HIGH RISK OF CARIES IN THE CITY OF BADALONA (BARCELONA)

**Authors:** A. García Pereiro, J. López Acedo, A. Gallardo Del Cosso, S. López Truchero, V. Muñoz Bollas, C. Nevot González

**Affiliations:** 1 CAP Apenins-Montigalˆ; Universitat de Barcelona, Badalona; 2 CAP Martí i Juliá, Badalona; 3 CAP Montgat-Tiana, Badalona; 4 CAP Progés-Raval, Badalona; 5 Universitat de Barcelona, Barcelona.

## Introduction

Epidemiological data in our country show a clear social gradient in dental caries among the child population, with greater prevalence and severity of lesions in children from more disadvantaged backgrounds.

## Objectives

To analyze the relationship between the socioeconomic level of neighborhoods and the percentage of children at high risk of cavities in the area where Badalona Serveis Assistencials, a publicly owned municipal organization, acts as the primary care provider.

## Material and Methods

The percentage of high-risk children was obtained from school oral health check-ups of first-grade students carried out by primary care dentists. The Òhigh-riskÓ classification is based on the criteria of the Department of Health of the Government of Catalonia, applied to children with a dmft (decayed, missing, and filled teeth in primary dentition) or DMFT (in permanent dentition) index greater than 0. The Territorial Socioeconomic Index (IST) is a composite index published by the Statistical Institute of Catalonia that summarizes the socioeconomic characteristics of small geographic areas.

## Results

A total of 860 seven-year-old schoolchildren were examined, of whom 35.93% (n=309) presented a high risk of cavities, with variability among Basic Health Areas (ABS) ranging from 48.35% in ABS Badalona-10 to 25.34% in ABS Montgat-Tiana. The Pearson correlation coefficient shows a strong negative correlation (-0.7647) between the percentage of high-risk schoolchildren and the Territorial Socioeconomic Index (IST) of their area of residence. When the outlier corresponding to ABS Badalona-8 is excluded, the coefficient rises to -0.9818, indicating a very strong correlation. However, methodological variables, particularly those related to the lack of diagnostic calibration, should be taken into consideration.

## Conclusions

The results show a high correlation between the neighborhood’s Territorial Socioeconomic Index and the percentage of 7-year-old schoolchildren at high risk of cavities. The results also show some limitations, the most important of which would be the lack of calibration among the examiners.

# Abstract: 03. IMPLEMENTATION OF DENTAL ASSISTANCE FOR THE POPULATION AGED 0 TO 6 YEARS IN THE BASQUE COUNTRY

**Authors:** N. Manuel Bejarano, I. Madariaga De La Fuente, M.V. Garcia Repilado, J.R. Fernández Ruiz

**Affiliations:** 1 Osakidetza- Dirección General, Vitoria-Gasteiz; 2 Osakidetza- Dirección General, Bilbao; 3 Osakidetza- Dirección General, Donosti

## Introduction

The latest national and regional epidemiological studies confirm a high prevalence of cavities in primary dentition in children aged 5-6 years and low dental visits. In line with WHO recommendations and the expansion of services offered by the National Health System, the development of a dental program for the 0-6 year old population was proposed in 2023, including caries risk assessment and the implementation of preventive measures.

## Objectives

Establish the foundations for this new service in the Basque Public Health System, develop/expand the technical and human infrastructure and inform the population of its implementation.

## Material and Methods

Coordinated by the Osakidetza Community Dental Service, interventions were carried out at various levels in 2023, including the expansion, equipping, and training of oral health units; establishment of referral pathways from Midwifery (Pregnancy Oral Health Program), Pediatrics (Child Health Program), and Administrative Departments. In addition, a new corporate act was created in agenda and content was incorporated into the Computerized History. Finally, the public was informed about the new service.

## Results

Implementation of a preventive oral care plan for the 0-6 age population, which became effective in November of last year.

## Conclusions

Currently, Osakidetza offers early interdisciplinary oral care for individual and collective benefit, aiming to eliminate inequalities in access to treatment in the most disadvantaged sectors. Furthermore, it offers a preventive environment from before birth, thus complementing public oral care in the Basque Country up to the age of 15.

# Abstract: 04. COMPREHENSIVE CLINICAL APPROACH TO DENTAL WHITE LESIONS. DIFFERENTIAL DIAGNOSIS, TREATMENT AND EVOLUTION

**Authors:** S. Prin Prin, A. Rodríguez Veiras, M.R. Garcillán Izquierdo

**Affiliations:** UCM, Madrid

## Introduction

In the following clinical case, the diagnosis, treatment, and follow-up of a patient with multiple pathologies are described. Generalized white lesions were found in various locations, such as the occlusal and gingival thirds. On the occlusal third, the enamel is rough and soft, with areas of wear and a "frosted" tooth appearance. A differential diagnosis was made among various enamel anomalies, such as dental fluorosis and amelogenesis imperfecta, concluding that the lesions are compatible with the hypomature form of amelogenesis imperfecta. The lesions located in the gingival third present a linear shape, which, due to their location and their relationship with dental biofilm deposits, are identified as incipient caries lesions, known as white spot lesions.

## Objectives

The goal of this clinical approach is to establish a comprehensive treatment plan and follow up to reflect objective changes in the level of enamel desmineralization.

## Material and Methods

The preventive protocol for treating white spot lesions includes the implementation of oral hygiene practices, such as brushing twice a day for two minutes with a fluoride toothpaste containing 1450 ppm, dietary changes, and remineralization with 22,600 ppm fluoride varnish applied for three consecutive weeks every three months. Two cycles of remineralization were performed.

## Results

To monitor the progression of demineralizations, the complementary diagnostic tool DIAGNOdentpen2190¨ was used for a follow up period of five months. During this period, a significant objective improvement was observed, as shown in the corresponding tables.

## Conclusions

The minimally invasive treatment of initial caries lesions is essential to prevent their progression. All changes can be monitored using tools such as the DIAGNOdentpen2190®.

# Abstract: 05. RADIOGRAPHIC EVALUATION OF BONE-RELATED LESIONS OF THE JAWS: A RETROSPECTIVE OBSERVATIONAL STUDY

**Authors:** S. Shabaka Fernández, P. Llabrés Catalá, D. Paz Vallejos

**Affiliations:** 1 Universidad de las Islas Baleares (UIB), Palma de Mallorca

## Introduction

Epidemiological studies of bone lesions aim not only to determine their frequency and distribution but also to identify factors influencing their occurrence, with the goal of improving their understanding and performing effective preventive strategies.

## Objectives

To describe the radiographic characteristics and prevalence of bone lesions in the jaws.

## Material and Methods

A retrospective observational study was conducted at the ADEMA university clinic. A total of 2,324 panoramic radiographs taken between 2018 and 2022 from patients aged 6 to 65 years were analyzed. From these, 527 were selected based on inclusion criteria: complete clinical records, high-quality radiographs without distortion, and no history of orthognathic surgery or maxillofacial trauma. Demographic and radiological data were collected, and lesions were evaluated based on location, shape, size, borders, radiodensity, and their effect on adjacent structures.

## Results

A total of 29.9% of patients presented with at least one bone lesion. Periapical lesions of inflammatory origin were the most common (79.1%). The mandible was the most frequently affected area (71.43%). No significant gender differences were observed, although males tended to present with multiple lesions. Prevalence increased with age, being highest in the 50–65 age group.

## Conclusions

This study reveals a high prevalence of bone lesions in the analyzed population, particularly those of inflammatory origin, highlighting the need for preventive measures and early detection. Aging appears to be a relevant risk factor, thus periodic radiographic monitoring in the adult population is recommended.

# Abstract: 06. THE ROLE OF THE DENTAL TEAM IN THE CHILDHOOD OBESITY PREVENTION AND MANAGEMENT PROGRAM IN CATALONIA (POICAT)

**Authors:** J. López Acedo, A. García Pereiro, T. Gallardo Del Cosso, S. López Truchero

**Affiliations:** 1 Badalona Serveis Assistencials (CAP), Badalona.

## Introduction

POICAT is a childhood obesity prevention and treatment program in Catalonia to reduce childhood overweight and obesity, with a comprehensive approach to health.

## Material and Methods

A pilot program was implemented between 2021 and 2022 in the La Mina neighborhood (Sant Adriˆ del Bes˜s), with the participation of families and local organizations. The intervention combined community and individual actions (care in health centers with multidisciplinary teams). Habits such as diet, sleep, physical activity, screen use, and positive parenting were addressed. The model is being extended to 35 Basic Health Areas with a high prevalence of obesity and vulnerability. The Montigalˆ-Badalona 9 center, where we work as dentists, was one of the 35 selected centers.

## Objectives

We believe dentistry has an important role in this program, considering that childhood obesity and caries share clear causal factors, such as poor diet and social vulnerability. Monitoring the oral health of these patients is a challenge; strengthening preventive measures will help reduce the prevalence of caries in our child population.

## Results

Childhood obesity was reduced from 26.4% to 25.4%. A notable decrease in sugary beverage consumption (from 68.6% to 19.6%) and an increase in healthy habits were observed.

## Conclusions

The program has proven to be effective. Its comprehensive, family-oriented, and non-stigmatizing approach, based on community and individual interventions, allows for a sustainable and scalable approach to addressing childhood obesity throughout the country.

# Abstract: 07. EVALUATION OF MICROLEAKAGE IN SEALANTS APPLIED WITH OR WITHOUT AN ADHESIVE AGENT: AN IN VITRO STUDY

**Authors:** J.E. Iranzo Cortés, M. Díaz Muñoz, T. Almerich Torres, C.F. Márquez Arrico, M. Puig Silla, J.M. Montiel Company

**Affiliations:** 1 Universitat de València, Valencia.

## Introduction

Dental sealants are a basic strategy in caries prevention, especially on susceptible occlusal surfaces. Their effectiveness depends, among other factors, on the application protocol.

## Objectives

To determine whether the use of an adhesive improves the marginal adaptation of the sealant and to assess whether adhesive pre-treatment enhances the long-term durability of the sealant.

## Material and Methods

An in vitro study was conducted on 38 sound human molars, which were randomly assigned to two groups. A resin-based sealant (Clinpro™ Sealant) was applied with and without an adhesive (Scotchbond™ Universal Plus) according to a standard acid-etching and light-curing protocol. The specimens were subjected to 500 thermocycling cycles, after which retention was evaluated using a probe. Following mesiodistal sectioning of the teeth, marginal leakage was assessed by dye penetration and microscopic analysis, with leakage recorded as absent or present in thirds of depth. Application times were adhered to the manufacturer’s recommendations to ensure reproducibility. The study protocol was approved by the Ethics Committee of the Universitat de València (Protocol No. 2024-ODON-3738564). Descriptive statistics and the chi-square test were performed for group comparisons (chi-square test, p < 0.05).

## Results

The adhesive group exhibited 100% complete retention, compared to 89.5% in the non-adhesive group. Both groups had 36.8% of specimens with no marginal microleakage. No significant differences in retention or marginal microleakage were observed between the two groups.

## Conclusions

The use of a prior adhesive did not result in a statistically significant improvement in sealant retention or marginal microleakage.

# Abstract: 08. IDENTIFYING REFERENCE GENES FOR RT-qPCR NORMALIZATION IN CHILDREN WITH DENTAL CARIES

**Authors:** J.M. Petano Duque, G.D. Arboleda Toro

**Affiliations:** 1 Grupo de Investigación en Biología Oral Avanzada, Universidad de Antioquia, Medellín, Antioquia, Colombia.

## Introduction

Dental caries is a multifactorial and frequent disease in childhood that leads to enamel and dentin demineralization. Gene expression studies are essential for understanding its molecular basis, and proper normalization using stable reference genes is crucial for reliable results.

## Objectives

To identify suitable reference genes for gene expression analysis in children with different stages of dental caries.

## Material and Methods

Saliva samples were collected from 27 children aged 8 to 9 years, categorized into three groups: advanced caries, incipient caries, and healthy. The stability of a panel of candidate genes was assessed using the comparative ÆCt method, BestKeeper, NormFinder, and geNorm. The RefFinder tool was used to integrate the results.

## Results

The 18s RNAr gene showed the highest expression level, whereas B2M exhibited the lowest. ACTB was the most stable gene in both carious groups, while GAPDH was more stable in the healthy group. The geNorm algorithm recommended using at least two reference genes for accurate normalization. Overall, 18s RNAr, ACTB, and YWHAZ were the most stable genes across conditions.

## Conclusions

To the best of our knowledge, this is the first study to identify suitable reference genes for expression analysis in children, considering the type and severity of carious lesions. 18s RNAr and ACTB may be used for the normalization of gene expression in saliva samples from children with different dental conditions.

# Abstract: 09. CARIES RISK DETECTION IN THE 3-4 YEAR OLD POPULATION OF SANT ANDREU DE LA BARCA (BARCELONA)

**Authors:** V. Muñoz Bollas, C. Parés Puyol, I. Martínez Lizán, C. Nevot González, M. Schemel Suárez, A. García Pereiro

**Affiliations:** 1 Facultat de Medicina i Ciències de la Salut -Universitat de Barcelona, Barcelona; 2 Centro de Atención Primaria Sant Andreu de la Barca ICS, Sant Andreu de la Barca - Barcelona.

## Introduction

Dental caries in early childhood is a chronic disease that is highly prevalent in our child population. The CAMBRA protocol helps us to implement measures to prevent the appearance of new lesions and slow the progression of existing ones.

## Objectives

To determine the risk of caries in the population of 3- and 4-year-olds in Sant Andreu de la Barca during the 2024-2025 school year and to assess the proposals for prevention and control of the disease that need to be implemented according to the CAMBRA protocol.

## Material and Methods

This cross-sectional study on caries prevalence assesses the risk of caries in 174 children aged 3 and 4 who are enrolled in  I4 at schools in Sant Andreu de la Barca, through clinical examinations at school that allow for the screening of children at high risk of caries. Subsequently, in the clinic,  the CAMBRA questionnaire is administered to parents as part of the application of this  protocol in the clinic.

## Results

Of the 174 children examined, 74 were at high risk of caries, representing 27% of caries in our sample.

## Conclusions

Early school screening and the application of the CAMBRA protocol in this population of 3-4 year olds has made it possible to effectively identify children who need measures to reduce and control the caries rate in this population group with a high prevalence of caries in primary teeth at a very early age. By implementing this protocol, the percentage will increase in the future.

# Abstract: 10. RELATIONSHIP BETWEEN THE QUALITY OF LIFE OF SPANISH DENTISTS AND THE USE OF THE INTERNET

**Authors:** E.M. Rosel Gallardo, R. Anaya Aguilar, C. Anaya Aguilar, P. Anaya Barea, A. Baca García, A. Hernández-Díaz Fernández De Heredia

**Affiliations:** 1 Departamento de Estomatología. Universidad de Granada, Granada; 2 Departamento de Organización de Empresas y Marketing. Universidad de Málaga, Málaga; 3 Departamento de Psicología Básica. Universidad de Málaga, Málaga; 4 Universitat Oberta de Catalunya, Barcelona.

## Introduction

The use of the Internet to obtain health resources and information is a very common practice and it allows tasks to be carried out with greater efficiency, precision and quality.  Some 68% of Spanish dentists use the Internet as an information tool in their working life. Several studies show that internet use can have both positive and negative effects on the quality of life of healthcare professionals.

## Material and Methods

A cross-sectional analytical study was carried out on Spanish dentists. To assess quality of life, an online survey was created using Limesurvey and sent via Mailchimp. The SF-36 v2 health questionnaire was included, assessing eight dimensions of health (four physical and four mental), summarized in two components: physical component and mental component.

## Results

Of the 568 surveys received from Spanish dentists, internet is used daily to chat (85%), to send and receive e-mails (76%), to search for information (68%), to read news (60%) and to visit social networks (51%). There is a positive correlation (rs ³ 0.09) between the frequency of internet use by dentists in Spain and their perception of quality of life in the physical component.

## Conclusions

Internet use has a significant impact on the quality of life of Spanish dentists. While moderate use of the Internet to search for information and read news can improve the perception of quality of life in the physical component, excessive use can have negative effects on the mental component.

# Abstract: 11. MY BRUSH MOVES TO THE RHYTHM OF A CHORUS: AN ORAL HEALTH PROMOTION STRATEGY

**Authors:** E. López De Castro De Castro, S. Ribeiro Grasa, X.D. Regra

**Affiliations:** 1 Clínica Conde Luna, Benavides de Orbigo; 2 Facultad de Medicina Dentaria, Lisboa; 3 Unidad Local de Salud de Beja, Beja (Portugal).

## Introduction

Promoting oral health in schools is key to establishing healthy habits from an early age. 

## Objectives

 Assess the impact of the ‘A Ritmo de Estrebillo Se Mueve Mi Cepillo’ oral health campaign developed for World Oral Health Day 2025.

## Material and Methods

Qualitative study design using focus groups with semi-structured interviews to a convenient sample of 20 parents and 20 teachers before and after the campaign. This playful educational strategy conducted in seven HIDES communities by dental hygienists actively involved 368 1st grade school children and consisted of a story, a song, and a parade.

## Results

The campaign was well received in the school community and improved message retention and attitudes of children and parents’ perceptions toward oral health. Parents report that children brush their teeth more often (85,7%), use less toothpaste (57,1%) and teachers report that more children started brushing their teeth after lunch in school (42,8%). Although parents have not reported any changes in their children's consumption of sweets, they have noticed that they brush their teeth more often after consuming sugar (57,1%). At school, teachers report an increase in fruit consumption during school snacks (28,5%).

## Conclusions

The identified strategy can serve as a basis for future interventions through community and educational approaches that involve teachers and families and create a favorable environment for children to maintain their oral health.

# Abstract: 12. TEENAGERS IN MALLORCA WITH PERIODONTITIS HAVE AN UNBALANCED NUTRIENT INTAKE

**Authors:** P. Estebala Alández, I. Coll Campayo, D.P. Vallejos Rojas, M.L. Bonet Piña, N. López Safont

**Affiliations:** 1 Universitat de les Illes Balears, Palma.

## Introduction

Adolescence is a key period for establishing dietary habits that will influence health throughout life. Oral health, which is closely linked to nutrition, is compromised by nutritional imbalances characterized by poor food choices. Literature reviews suggest an association between malnutrition and a higher prevalence of oral disease in adolescents. In this context, our study aims to analyze the relationship between low intake of nutrients protective against periodontitis and the development of the disease in the adolescent population.

## Objectives

The main objective of this project is to identify a possible association between malnutrition—specifically a low intake of nutrients related to good oral health—and periodontal disease in the adolescent population of Mallorca.

## Material and Methods

A total of 178 15-year-old adolescents were analyzed and classified according to their oral health status (healthy vs. diseased). A 24-hour dietary recall was collected to assess nutrient intake using the Nutrium nutrition software, in order to study the relationship between malnutrition and the presence of periodontitis. Nutritional data were analyzed using the SPSS statistical program.

## Results

Significant differences were found between adolescents with and without periodontitis in their intake of vitamin A (healthy: 667.81 ± 97.06 vs. diseased: 334.34 ± 29.32; p = 0.004), with no significant differences observed for other nutrients or total energy intake.

## Conclusions

Adolescents with lower vitamin A intake have a higher prevalence of periodontitis, which reinforces the importance of ensuring adequate vitamin A consumption to maintain good oral health.

# Abstract: 13. PRELIMINARY FINDINGS FROM A COMMUNITY BASED INTERVENTION TO PREVENT EARLY CHILDHOOD DENTAL CARIES

**Authors:** N. Bernardo Vilamitjana, L. Figueroa Marcé, Z. Bastardo López, E. Olabarrieta Zaro, M. Pujiula Blanch

**Affiliations:** 1 Centro de Atención Primaria Alfons MorŽ, Salt, SALT.

## Introduction

Urban municipality with 33,337 inhabitants, 38% immigrant population, and low socioeconomic status. High prevalence of childhood caries reported by the Public Health Agency, a community health program was launched to improve oral health.

## Objectives

To prevent the onset of caries in children under 3 years old from families at high risk of dental decay.

## Material and Methods

Patient recruitment was carried out through Primary Care consultations in pediatrics, dentistry, and dental hygienist. The intervention took place in the dentist and hygienist’s office and included an oral examination, toothbrushing techniques, and dietary advice. Follow-up was recommended every 3, 6, or 12 months depending on caries risk. Families were invited to participate in three multidisciplinary group sessions (nutritionist, emotional well-being specialist, and dental hygienist) aimed at improving hygiene and dietary habits. Results will be compared with those from a population with similar characteristics.

## Results

In 2023, the dental service examined 71 children under the age of 3. Of these, 67 were caries-free and 4 had caries. In 2024, 41 of the 67 caries-free children were followed up, and 3 new cases of caries were diagnosed. Only one of these cases showed progression of lesions in 2025. Children diagnosed with caries in 2023 did not present new lesions. A total of 19 families participated in the group activities, with good acceptance and engagement.

## Conclusions

 Preliminary evidence suggests that the interventions contributed to reducing the incidence of caries. Completion of the comparative study will be necessary to assess the effectiveness of the multidisciplinary community intervention.

# Abstract: 14. DOCTORAL THESES IN DENTISTRY: A STUDY OF SPANISH PRODUCTION BETWEEN 2020 AND 2024

**Authors:** M.V. Eustaquio Raga, A. González Teruel, M. Bolaños Pizarro, J.M. Montiel Company, C.F. Márquez Arrico

**Affiliations:** 1 Facultat de Medicina i Odontologia, Universitat de València.

## Introduction

Doctoral theses represent the highest degree of scientific and academic expression, and are individual, unpublished research works that make an original contribution.

## Objectives

Analyze the scientific production of Spanish theses in dentistry.

## Material and Methods

This is a retrospective, cross-sectional study whose primary source of information consisted of doctoral theses in Dentistry defended from January 2020 to December 2024. The database used was Teseo. Additionally, Dialnet-Tesis and various university repositories were used to complement the information provided by Teseo. The following variables were collected: title and author, gender, university, date, topic and type of study, descriptors, and thesis directors.

## Results

Out of a total of 439 theses/authors, 41.2% were men and 58.8% were women (87.8 theses/year). We obtained 451 thesis advisors (278 men and 173 women). The Complutense University of Madrid (18.4%) accounted for the largest number of theses defended per university in the 5 years studied, followed by the University of Seville and the University of Valencia (14.3% and 14.1%, respectively). A total of 396 were presented at public universities. 21.7% of theses chose dental implants as their main topic, and 7.5% chose systematic reviews/meta-analyses as their study type.

## Conclusions

This study reveals a progressive increase in the number of women authoring doctoral theses. The wide variety of topics and methods is notable, as is a predominance of public universities and dental implants.

# Abstract: 15. UNILATERAL MASTICATION EXPLAINED FROM A NEURO-OCCLUSAL REHABILITATION PERSPECTIVE

**Authors:** V. Balmaseda idal, Y. Allouch Azirar, H.S. Babani Babani, M. Alonso Ongil, A.J. López Jiménez

**Affiliations:** 1 Universidad Complutense de Madrid, Madrid.

## Introduction

Neuro-Occlusal Rehabilitation is a clinical and preventive discipline in dentistry that focuses on the early identification and correction of stomatognathic functional and morphological imbalances. At the same time, unilateral mastication is a noxious habit based on the parafunctional rhythmic movement of mastication on one side, either exclusively or preferentially.

## Objectives

Explain unilateral mastication's development, its associated morphological and functional changes in the stomatognathic system, and justify its treatments based on NOR's laws and parameters.

## Material and Methods

A search was conducted to identify studies, systematic reviews, and textbooks about NOR, unilateral mastication, and their relationship. It was done through PubMed, Cochrane, and classic literature with the terms "NOR" OR "CROSSBITE".

## Results

 Unilateral mastication leads to different changes in the mandibular ramus and condyle, the maxilla, and masticatory muscles in the working side compared to the balancing side. The changes altogether induce the development of a crossbite. The FMAP and NOR laws serve as the basis for preventive and interceptive treatments, such as selective cuspal reduction, coronoplasty, occlusal bite ramps, and occlusal guides.

## Conclusions

NOR not only provides predictive parameters for crossbite and its treatments but also justifies the effect of parafunctional masticatory activity on occlusion and musculoskeletal stomatognathic structures.

# Abstract: 16. INTERPROFESSIONAL EDUCATION AND HEALTHY HABITS: EXPERIENCE FROM THE 13TH ORAL HEALTH WEEK AT UEM

**Authors:** J. Sánchez Ituarte, M. Frías Senande, S. Peinado Escalada, L.J. Marcos Zambrano, H. Marcos Pasero

**Affiliations:** 1 Universidad Europea de Madrid, Villaviciosa de Odón.

## Introduction

Oral health is fundamental during university years, as habits acquired at this stage can have lasting effects. As part of the ÒHealthy CampusÓ initiative, faculty members from Universidad Europea de Madrid developed an interdisciplinary project with students from Dentistry, Human Nutrition and Dietetics, and Oral Hygiene to promote oral health from a comprehensive perspective.

## Objectives

To promote and assess healthy habits through an interprofessional educational intervention and a survey, raising awareness about the risks of hidden sugar consumption and oral hygiene.

## Material and Methods

During the 13th Oral Health Week (March 2025), the 250 participating students organized workshops, interactive stands, distributed samples, and conducted surveys. Statistical analyses were performed using R (version 4.5.1). Differences between groups were evaluated using Fisher’s exact test or Pearson’s Chi-square test, according to statistical assumptions.

## Results

The sample included 436 subjects, mostly women (66%) and aged 16 to 25 years (84%). Comparing students from health-related faculties (N = 350) with those from other faculties (N = 86), no significant differences were found in brushing frequency (p = 0.15) or type of toothbrush used (p = 0.6). However, significant differences were observed in the use of fluoride toothpaste (p = 0.002) and in the frequency of dental visits (p = 0.013), both higher among health-related students.

## Conclusions

The intervention promoted awareness of oral health and nutrition. Furthermore, interprofessional collaboration enriched the students’ learning experience.

# Abstract: 17. PREVENTION OF RISK FACTORS FOR PERI-IMPLANT DISEASES

**Authors:** A. Sharif rdestanian Moghadam, H. Habibollahi, M. Amirbagloy Darian, J.F. Martín Morales

**Affiliations:** 1 Universidad Complutense, Madrid.

## Introduction

The placement of dental implants can lead to peri-implant complications which also affect oral health. Prevention is key and should be based on personalized approach that considers both local and systemic factors,particularly the controllable ones. In dental practice, it is essential to establish educational and motivational programs for patients with implants to minimize these pathologies.

## Objectives

To identify local and systemic risk factors that may contribute to peri-implant disease,as well as preventive measures to avoid them.

## Material and Methods

A literature review of PubMed-indexed articles published between 2018 and 2025.

## Results

Local factors, such as anatomical, surgical, and prosthetic conditions, complicate hygiene and promote bacterial plaque accumulation. Relevant systemic factors include diabetes, metabolic syndromes, cardiovascular diseases, and harmful habits such as smoking.  Post-functional loading risk factors include inadequate prosthetic design or surgical planning, whereas reduced mucosal thickness and limited width of keratinized mucosa represent significant risk factors for dehiscences. Oral hygiene plays a crucial role in the prevention of peri-implant diseases, along with adequate supracrestal height, a favorable restorative profile as part of the prosthetic design, and sufficient thickness and width of the peri-implant mucosa.

## Conclusions

Mucositis, peri-implantitis, and dehiscence are the most common complications. Standardized protocols should be developed to prevent these conditions.

# Abstract: 18. NEW ADVANCES IN REMINERALIZATION

**Authors:** J. Fernández Patrón, B.L. García Rovira

**Affiliations:** 1 Universidad Complutense de Madrid, Madrid.

## Introduction

The demineralization of enamel and dentin, induced by bacterial acids, promotes the development of caries and dental sensitivity. In response, remineralization constitutes a non-invasive strategy to reverse early lesions. Although fluoride is the therapeutic standard, biomimetic alternatives with the potential to complement or replace its action have emerged over the past decade.

## Objectives

To evaluate techniques and materials for the remineralization of early carious lesions, with an emphasis on non-fluoride agents.

## Material and Methods

A search was conducted in PubMed and Cochrane for articles published between 2015 and 2025, in English and Spanish, with open access. Included were human clinical trials, systematic reviews, and meta-analyses focused on the remineralization of incipient lesions. Excluded were purely in vitro studies, articles published before 2015, paywalled content, and studies focused solely on fluoride. Out of 1,340 initial records, 18 articles were selected.

## Results

 Eight studies evaluated fluoride compounds, eight examined ACP (amorphous calcium phosphate), three investigated nanohydroxyapatite, and two studied self-assembling peptides. ACP combined with casein phosphopeptides (CPP-ACP) showed efficacy comparable to or greater than fluoride. Nanohydroxyapatite showed promise in both enamel and dentin. The P11-4 peptide demonstrated the ability to induce remineralization through nanofibrillar scaffolds. However, much of the current evidence still stems from limited studies.

## Conclusions

Biomaterials such as CPP-ACP, nanohydroxyapatite, and self-assembling peptides represent viable biomimetic alternatives to fluoride. Nevertheless, randomized and long-term clinical trials are needed to validate their efficacy and safety.
